# Development of a Battery-Free, Chipless, and Highly Sensitive Radio Frequency Glucose Biosensor

**DOI:** 10.3390/mi15020272

**Published:** 2024-02-14

**Authors:** Md. Rajibur Rahaman Khan

**Affiliations:** Research Institute of Engineering and Technology, Hanyang University, Ansan 15588, Republic of Korea; rajibur@hotmail.com

**Keywords:** glucose biosensor, phenylboronic acid, dielectric constant, interdigitated capacitor, resonance frequency

## Abstract

In our study, we designed and developed a glucose biosensor that operates without a battery or chip. This biosensor utilizes the principles of radio frequency (RF) to operate. For the construction of a glucose-sensitive interdigitated capacitor (IDC), a famous glucose-sensitive substance called phenylboronic acid (PBA) is combined with a polyvinyl chloride (PVC) and n,n-dimethylacetamide (DMAC) solution. According to the theory of radio frequency sensing, the resonance frequency shifts whenever there is a change in the capacitance of the glucose-sensitive IDC. This change is caused by the fluctuations in glucose concentrations. As far as we are aware, this is the first glucose sensor that employs the RF principle to detect changes in glucose solution concentrations using PBA as the principal glucose-sensitive material. The sensor can detect glucose levels with remarkable sensitivity, around 40.89 kHz/decade, and a broad dynamic range covering 10 μM to 1 M. Additionally, the designed biosensor has excellent linearity performance, with a value of around 0.988. The proposed glucose biosensor has several benefits: lightweight, inexpensive, easy to build, and an acceptable selectivity response. Our study concludes by comparing the proposed RF sensor’s effectiveness to that of existing glucose sensors, which it outperforms.

## 1. Introduction

Diabetes mellitus is widely recognized as one of the most detrimental diseases to the human body over an extended period. In cases when the pancreas is unable to produce sufficient insulin to regulate blood sugar levels or when the body is unable to make effective use of the insulin that is produced, this illness can develop [1]. Diabetes may result in a variety of additional medical issues if it is not correctly managed. These diseases include kidney disease, hypertension, heart disease, stroke, the possibility of causing complications during pregnancy, eye complications, foot complications, nerve damage, hyperglycemic non-ketotic syndrome, mental health disorders, and gastroparesis [2,3,4]. In this situation, a glucose sensor can help detect glucose levels in the human body so that they can take proper treatment or be careful before developing severe health problems. Thus, many researchers have tried improving glucose sensing performance in recent decades based on the material, different structures, and various sensing techniques [3,4,5,6,7,8,9,10,11,12].

Many bodily fluids, such as blood, saliva, tears, and sweat, contain glucose, and glucose may be detected or found in these fluids. The eccrine glands are responsible for the production of a fluid that is often referred to as sweat. A wide range of chemical groups may be found in sweat, and these substances are useful indicators for monitoring one’s health and determining diagnoses. A wide variety of chemical components, including Na^+^, K^+^, Ca^2+^, Cl^−^, hormones (cortisol), proteins (IFN-γ), peptides, and numerous metabolites, including lactate, glucose, and alcohol, are present in sweat. These substances are essential indicators for diagnosing sickness and observing health [1,2]. The amount of glucose in human sweat ranges from 0.06 to 0.2 mM, which is equivalent to 3.3 to 17.3 mM in blood glucose for humans [2].

Yue Lin et al. developed a multicolor, colorimetric glucose biosensor [5]. By etching gold nanorods (AuNRs) with TMB^2+^ oxidation, the researchers were able to utilize the optical properties of AuNRs in their investigation. The color of the AuNRs changed when the concentration of glucose changed. The sensor has several advantages, including simplicity of use, low production costs, quick response times, and the capacity to change color in response to changes in glucose concentration that are visible to the naked eye. The primary drawback of this sensor is that it has a limited glucose measurement range, which is between 0.1 and 1.0 mM. Mahadeva and Kim proposed a conductometric glucose biosensor using a hybrid nanocomposite made of cellulose and tin oxide [6]. The sensor has a modest detection range of around 0.5 to 12 mM, even though it has high linearity-related performance.

Zhai et al. presented a glucose sensor that utilizes carbon dots and a fluorophore (m-dihydroxybenzene). The sensor operates on the principle of fluorescence ratiometry [7]. The sensor possesses a high level of detecting capability, linear performance, and exceptional selectivity, yet it is characterized by a limited dynamic range that spans around 10 to 200 μM. Zhang et al. designed a plasmonic probe-type sensor using a single-mode optical fiber to measure pH and glucose [8]. The researchers applied a thin layer of silver film, measuring in nanometers, onto the slanted fiber Bragg grating. Additionally, they placed a layer of gold film on the tip of the optical fiber probe. The sensor’s construction and working concept are straightforward. Nevertheless, there are some drawbacks associated with this method. These include a difficulty in determining the resonance wavelength, the need for large and costly apparatus to measure wavelength changes, a limited glucose detection range of 0 to 12 mM, and a relatively long reaction time of around 20 min.

A passive LC (inductor-capacitor) radio frequency (RF) sensor functions similarly to passive RFID tags, but its purpose is to detect or measure specific physical or chemical characteristics rather than just transmitting an identifier [13]. These sensors utilize the same fundamental components, such as inductors and capacitors, to perceive alterations in the surroundings and respond accordingly. Passive LC RF sensors are beneficial in situations where they are crucial to function wirelessly and without the need for batteries. They are utilized in many sensing applications, including environmental monitoring, industrial process control, healthcare, and Internet of Things (IoT) deployments [14]. Because they do not have an internal power supply, they are well suited for applications that prioritize compactness, cost-effectiveness, and very little maintenance [13,14,15].

Tiwari et al. presented a glucose-detecting, biodegradable, flexible RF sensor [9]. The sensor is straightforward to fabricate and exhibits linear performance throughout its glucose measurement range. On the other hand, the sensor can only detect glucose between 0 and 80 mM, and its selectivity is poor because the sensor’s interdigitated capacitor (IDC) does not use any glucose-selective material or a method for detecting selectivity. Shaffat et al. created a glucose-to-resistance transduction sensor that does not require a battery or a chip [10]. The sensor’s fabrication and sensing mechanism are straightforward. Additionally, the sensor has satisfactory linearity performance. However, a poor dynamic range of 0 to 12 mM and a lengthy response time limit it.

In our study, we proposed an RF biosensor/sensing element that is a parallel combination of an IDC and a planar inductor. A phenylboronic acid (PBA)–polymer matrix is used as a dielectric material in the glucose-sensitive IDC. When the glucose solution comes into contact with the dielectric material of the IDC, its capacitance changes, and as a result, the resonance frequency of the sensor changes. Generally, the sensor that is based on the IDC only and whose variation in capacitance corresponds to the variation in voltage across the capacitor cannot detect tiny capacitance variations for very low concentrations of glucose in solution. For detecting tiny capacitance variations, lots of sensing methods are available; one of them is the RF method. Since the proposed sensor is based on the RF sensing principle and its resonance frequency is selected in the MHz range, for a tiny capacitance change, its resonance frequency changes. Thus, the proposed sensor is highly sensitive to detecting low to high concentrations of glucose. Using a portable VNA, the resonance frequency can be easily measured. The proposed sensor has many features, such as being easy to fabricate, low cost, highly sensitive, linear sensing performance, excellent reproducibility performance, highly stable, etc. Lastly, in our study, we observed the performance of the proposed sensor compared to other glucose sensors, and we found that the overall response of the proposed sensor is better than the others.

## 2. Theory and Operational Principle

**Figure 1:**
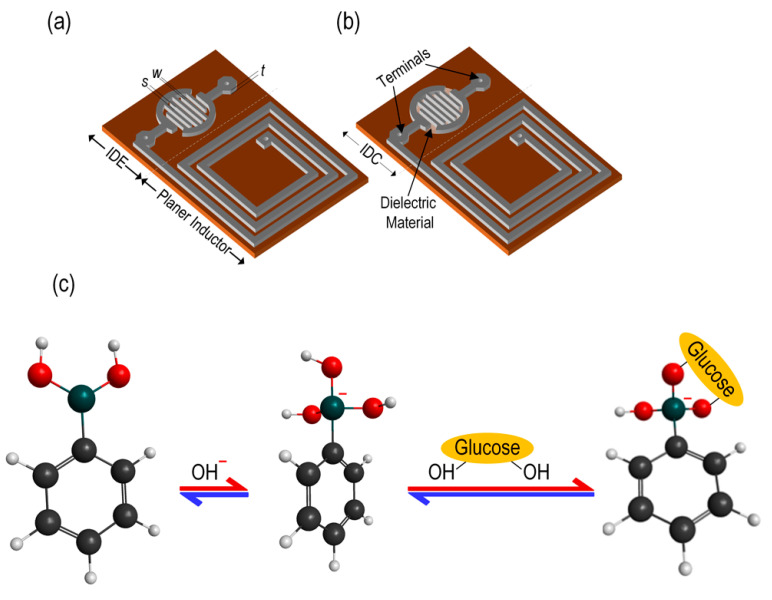
(**a**) Schematic of the RF glucose sensor without a sensing membrane in the IDE; (**b**) schematic of the RF glucose biosensor with a sensing membrane in the IDE to form the IDC; and (**c**) the molecular structure of phenylboronic acid and its reaction mechanism with glucose.

In this work, we utilized an IDC and a planar inductor to prepare an RF-based glucose biosensor. Figure 1a,b shows the schematics of the IDE with a planar inductor on the same board and the proposed glucose biosensor, respectively. We used phenylboronic acid (PBA), classified as a meso-Lewis acid and a polymer, to prepare the IDC’s glucose-sensitive dielectric material. In water-based environments, PBAs and hydroxide ions react to form boronates. The concentration of hydroxide ions determines how many boronates are formed. To put it another way, boronate formation completely depends on the pH. The glucose’s hydroxyl groups produce a cyclic ester, which is then consumed when combined with the boronate form. As a consequence of this, the incorporation of glucose results in a reduction in the boronic acid form, which in turn leads to a drop in the apparent pKa [11,12]. The reaction is presented in Figure 1c. A drop in the pKa of an organic acid occurs when the dielectric constant is increased, and vice versa [12].

Now, if *l* is the average length of the finger, *w* is the width of the finger, *s* is the gap between two fingers, and *N* is the number of electrodes. The overall capacitance of the IDC may be determined using the following mathematical expression [15]:

(1) $$C_{IDC}=\frac{\varepsilon_{0}\left(1+\varepsilon_{r}\right)(N-1)l}{2}\left\{\frac{K\sqrt{\left(1-k^{2}\right)}}{K(k)\;}+\varepsilon_{0}\frac{t}{s}\right\}$$

where $\varepsilon_{0}=8.854\times 10^{-12}F/m$ is the free space permittivity and $\varepsilon_{r}$ is the dielectric constant of the PBA-containing polymer matrix of the IDC. K(k) is the first type of elliptic integral of the *k* modulus and is given by:

(2) $$k=\frac{w}{s}$$

and

(3) $$K=\left(1+\frac{2w}{2g+w}\right)\times \left(\sqrt{\frac{1}{1+\frac{2w}{g}}}\right)$$

Therefore, if the glucose concentration changes, then the capacitance of the IDC changes. Now, the inductance of the square-shaped spiral/planer inductor can be written as the following equation [16]:

(4) $$L=1.39\times 10^{-6}\left(d_{o}+d_{i}\right)n^{5/3}\log4\left(\frac{d_{o}+d_{i}}{d_{o}-d_{i}}\right)$$

In (4), *n* represents the number of turns that are present in the spiral inductor, while *d_o_* and *d_i_* represent the outer and inner diameters of the spiral, respectively. With the two components (*C_IDC_* and *L*) of the circuit linked in parallel, the sensor’s resonance frequency, *f_0_*, is given by [13]:

(5) $$f_{0}=\frac{1}{2\pi \sqrt{LC_{IDC}}}$$

It follows that the resonance frequency of the parallel *LC_IDC_* circuit reduces when the capacitance of the IDC increases, which corresponds to an increase in the glucose concentration, and vice versa.

## 3. Experiment Details

### 3.1. IDE and Planer Inductor Fabrication

**Figure 2:**
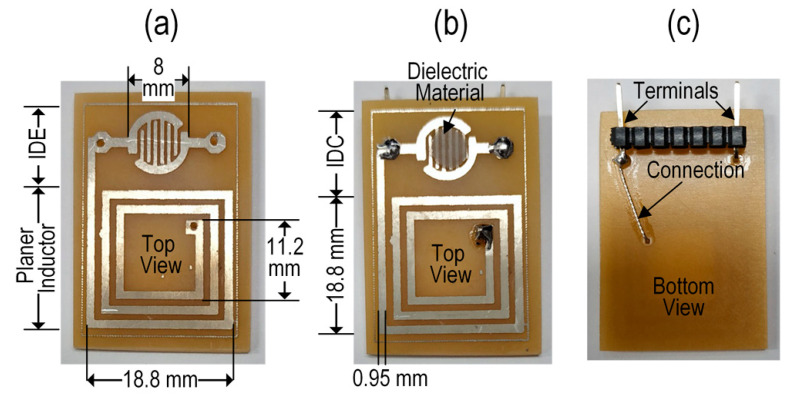
Photographs of the fabricated glucose biosensor. (**a**) A top view of the sensor before depositing the sensing membrane in the IDE; (**b**) a top view of the sensor after depositing the sensing membrane in the IDE to create the IDC glucose biosensor; and (**c**) a bottom view of the fabricated glucose biosensor.

In our research, we prepared a parallel combination of an IDE and a planar inductor on a copper board 20 mm × 30.5 mm in size. To design the IDE and planer inductor, we first deposited the photoresist film on the copper board, and then we placed the appropriate mask pattern on the board and applied UV light for 10 min. FeCl_3_ was applied to remove the copper section that was deemed unwanted from the board. Lastly, a N_2_ gas flow was used to dry the pattern board after cleaning it with deionized (DI) water. The IDE’s electrodes had a width (*w*) of approximately 0.40 mm and a spacing (*s*) of approximately 0.45 mm between adjacent electrodes. Figure 2a shows a photograph of the fabricated IDE and planer inductor on the same board.

### 3.2. Materials and Fabrication of the Glucose-Sensitive IDC

All of the following substances were acquired from Sigma-Aldrich: n,n-dimethylacetamide (DMAC), PBA, polyvinyl chloride (PVC), glucose, sodium chloride (NaCl), urea, lactic acid, and uric acid. Since all materials utilized were of analytical purity, it was not essential to complete any further purification procedures. The polymer PVC is a highly desirable material for membranes due to its rigidity; affordability; exceptional physical and chemical characteristics; ability to resist acids, alkalis, and solvents; and for its mechanical qualities [17]. Thus, PVC is commonly utilized for the production of high-performance membranes for this particular situation. On the other hand, DMAC is a natural substance represented by the chemical formula CH_3_CON(CH_3_)_2_. It is a widely available and cost-effective organic solvent that does not donate protons. As a solvent that is polar in nature, this water-miscible colorless substance with a high boiling point is frequently utilized in the process of organic synthesis. We employed DMAC as a solvent due to its classification as an aprotic solution, which ensures that it does not undergo any chemical reactions with other molecules.

PBA exhibits glucose sensitivity. We prepared the glucose-sensitive dielectric solution by combining 0.15 g of PBA with 2 mL of DMAC solution and sonicating the mixture for 10 min. Subsequently, 0.2 g of PVC was added to complete the solution. This dielectric solution was placed on an orbital agitator for seven days to ensure it became a homogeneous mixture.

The IDE of the sensing element was cleaned in stages with ethanol, methanol, and DI water. It was then dried under a N_2_ gas flow. After this, the dielectric sensing solution was applied to the IDE via drop casting. The IDE was converted into an IDC after depositing the dielectric solution onto it. To dry the wet IDC, the oven was set to 70 °C for 30 min. Photographs of the fabricated sensor/sensing element’s top and bottom views are shown in Figure 2b and Figure 2c, respectively. For the best IDC sensitivity, the electrode and sensing membrane thicknesses should be identical. The sensing membrane thickness may be estimated at 35 μM, matching the IDE thickness.

### 3.3. Experimental Setup of the Glucose-Sensitive IDC and Sensory System

The proposed sensor works on the resonance frequency modulation principle. The sensor is a parallel combination of a glucose-sensitive interdigitated capacitor (*C_IDC_*) and a planar inductor (*L*), called a parallel *LC_IDC_* resonant circuit. The capacitance of the IDC and the resonance frequency of this *LC_IDC_* resonant circuit (proposed glucose biosensor) are given in (1) and (5), respectively. the dielectric of the IDC consists of a glucose-sensitive material. So, the glucose concentration variation changes the glucose-sensitive IDC’s capacitance, corresponding with a change in the resonance frequency of the glucose biosensor. We used a VNA to observe the variation in the resonance frequency shift. If the concentration of glucose increased, then the capacitance of the IDC increased, and the sensor’s resonance frequency decreased, causing a red shift in the resonance frequency and vice versa.

**Figure 3:**
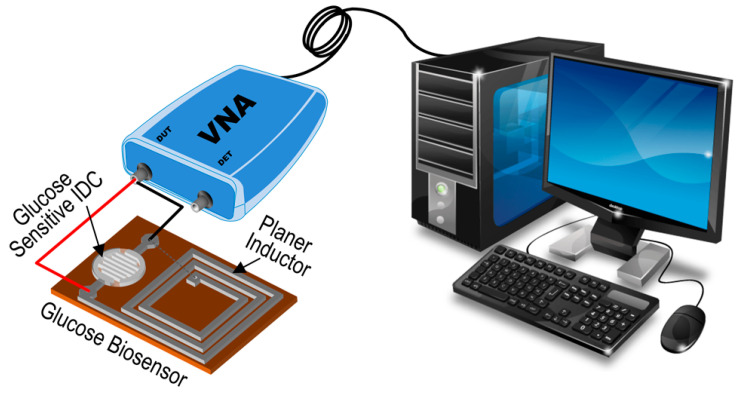
Schematic of the experimental setup to detect glucose.

A schematic of the experimental setup of the proposed glucose-sensing system is presented in Figure 3. First, we injected one drop of DI water as a reference solution to set the reference benchmark. Then, various doses of glucose solution were dropped onto the IDC of the sensing element to observe the resonance frequency shift due to the variation in different glucose concentrations. Contact between the glucose solution and the IDC’s sensing membrane or dielectric material altered the membrane’s dielectric constant, which influenced the capacitance of the glucose-sensing element. As a result, the resonance frequency shifts, which depends on the glucose concentration.

## 4. Results and Discussions

**Figure 4:**
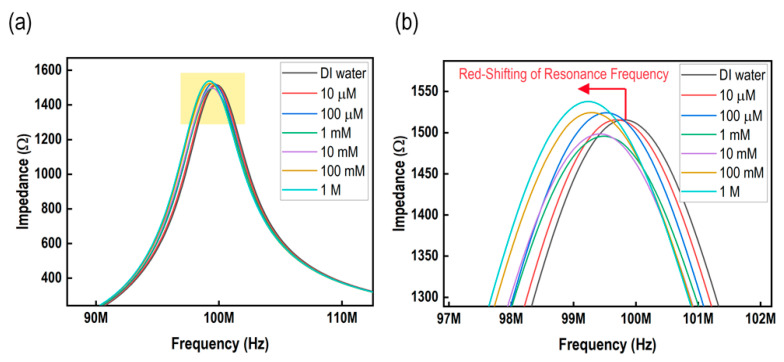
Performance of the sensor. (**a**) Frequency response of the sensor; and (**b**) enlarged view (indicated in yellow rectangular in (**a**)) of the frequency response of the sensor.

Figure 4a illustrates the shift in the resonance frequency as the concentration of glucose solution varies from 10 μM to 1 M. The resonance frequency values corresponding to these concentrations are around 99.75, 99.702, 99.68, 99.62, 99.595, and 99.54 MHz. The difference in the resonance frequency shift between the reference solution (DI water, resonance frequency 99.78 MHz) and the 1 mM glucose solution was about 99.7 kHz. Based on the data shown in Figure 4b, it can be observed that an increase in concentration leads to a red shift in the resonance frequency. The resonance frequency of the sensing element undergoes a red shift due to the increase in the dielectric constant of the IDC when the concentration of glucose increases. This increase in the dielectric constant leads to an increase in the capacitance of the sensing element.

**Figure 5:**
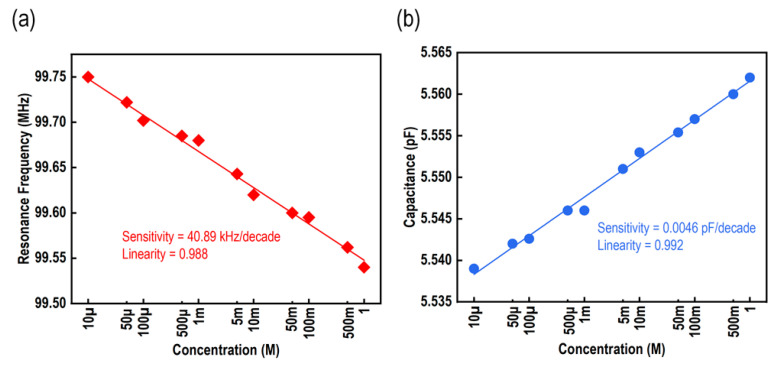
Performance of the sensor. (**a**) Resonance frequency vs. different concentrations of glucose solutions; and (**b**) capacitance vs. glucose concentration.

Figure 5a depicts the sensing response of the proposed glucose-sensing element across a range of concentrations, from 10 μM to 1 M. A linear relationship and a broad measurement range characterize the performance of the glucose-sensing element. There was a direct relationship between the glucose concentration and resonance frequency, meaning that as the concentration increases, the resonance frequency decreases, and vice versa. The designed glucose biosensing element exhibited a sensitivity of around 40.89 kHz/decade and a linearity of 0.988. The graph in Figure 5b illustrates the change in the IDC capacitance of the glucose-sensing element in relation to various glucose concentrations. The relationship between the two variables is linear. The IDC sensitivity of the proposed glucose biosensing element in terms of capacitance was around 0.0046 pF/decade, while the linearity was measured at 0.992.

**Figure 6:**
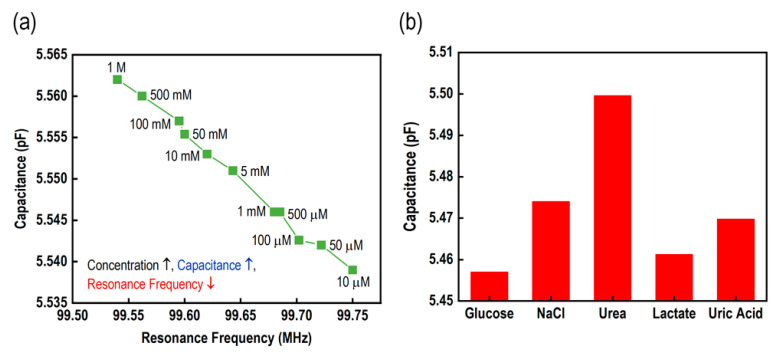
Performance of the sensor. (**a**) The relationship between capacitance and the resonance frequency under different concentrations of glucose; and (**b**) the selectivity response of the sensor under 1 mM of different solutions.

Figure 6a illustrates the correlation between the change in resonance frequency and the change in capacitance for a particular glucose concentration. From these results, it is evident that an increase in the concentration of glucose leads to an increase in the capacitance of the IDC. Consequently, this causes a decrease in the shift of the resonance frequency. In our proposed glucose biosensor, we used different biomaterials available in the swart. The biomaterials comprised NaCl, urea, lactate acid, uric acid, and a mixture of other substances. Figure 6b illustrates the proposed sensing element’s selectivity performance when applied to a variety of biomaterial solutions at a concentration of 1 mM. Based on the results of this study, we can say that PBA is a moderately selective material for glucose, and the designed biosensor can successfully distinguish glucose from other solutions.

The response time was determined by monitoring the increase in the IDC capacitance of the sensing element from 10% to 90% of its stable value at a certain glucose concentration. To fulfill this objective, we opted for a glucose solution with a concentration of 100 µM. Upon applying a glucose solution over the IDC of the glucose-sensing device, we observed that the capacitance fluctuation reached a steady state within a time frame of 3 s. Subsequently, we employed tissue paper to remove the glucose solution from the IDC. The sensor reached its equilibrium state within a time frame of 3 s. Hence, we may infer that both the sensor’s response and recovery times are under 3 s.

**Figure 7:**
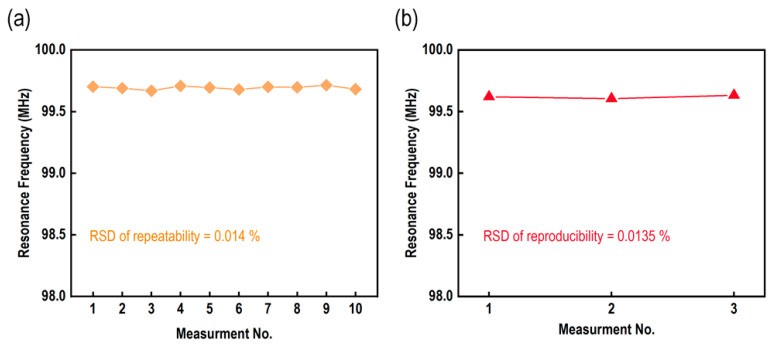
(**a**) The repeatability performance of the proposed glucose biosensor, and (**b**) the reproducibility performance of the proposed glucose biosensor.

Ten data values were taken into consideration to evaluate the repeatability and relative standard deviation (RSD) of the proposed passive RF glucose biosensor. The resonance frequencies of these values were 99.702, 99.69, 99.669, 99.708, 99.695, 99.679, 99.70, 99.697, 99.714, and 99.682 MHz, respectively, and the data are graphically presented in Figure 7a. A statistical metric that expresses the precision of a group of values is called the relative standard deviation (RSD), and repeatability is a term that describes the consistency of measurements when the same conditions are applied. In the context of this discussion, the recorded data points represent several measurements that were taken using the proposed passive RF glucose biosensor under conditions that were considered to be comparable. The measurements were taken at a concentration of 100 µM of glucose solution in this instance. To determine repeatability, we examined the degree to which these measurements cluster around an average. This provided an indication of the sensor’s precision and reliability when applied to comparable situations. The relative standard deviation (RSD) was relatively low, approximately 0.014%, indicating that the proposed RF glucose biosensor provided data that was trustworthy and consistent under the specified parameters. From Figure 7a, we can also see that no remarkable variation occurs within the ten measurements, and so we can conclude that the proposed glucose biosensor offers a stable response.

To observe the reproducibility performance, we prepared three samples of the proposed glucose biosensors and measured the resonance frequency at 10 mM glucose solution, as shown in Figure 7b. The resonance frequencies of the three measurements were about 99.62, 99.605, and 99.632 MHz, respectively. The RSD of the reproducibility was about 0.0135%. Therefore, we can say that the proposed glucose biosensor exhibited a good reproducibility performance. The specifications of the proposed RF glucose biosensor are listed in Table 1.

As part of our research, we compared how well the proposed RF glucose biosensor worked compared to different types of sensors, such as colorimetric, fluorescence, fiber grating, conductometric, RF, and electrochemical sensors. The dynamic range, linearity, and response/recovery time were some of the criteria that were considered. We determined that the performance of the developed sensor surpassed that of the ones mentioned above. The performance characteristics of several sensors are presented in Table 2.

## 5. Conclusions

In this study, we proposed an RF resonance frequency-shifting biosensor that can detect glucose from 10 μM to 1 M. A well-known glucose-sensitive material, namely phenylboronic acid-containing polymer matrix, was employed as a glucose-sensitive dielectric material in the interdigitated capacitor (IDC) of the sensing element. The proposed glucose bio-sensitive sensing element was a parallel combination of the IDC and the planer inductor. In this study, the resonance frequency shifted as the concentration of the glucose solution changed. The sensitivity and linearity of the proposed RF resonance frequency-shifting glucose biosensing element were about 40.89 kHz/decade and 0.988, respectively, with a fast response time of approximately 3 s. The sensing element exhibited moderate/acceptable selectivity, steady response, and exceptional repeatability. The proposed sensing element also exhibited several advantages, such as cost-effectiveness in fabrication, simplicity in the fabrication process, lightweight, and more. The performance of the proposed sensing element surpassed that of conventional sensors. We want to utilize this technology to create a VOC gas sensor in the future.

## Figures and Tables

**Table 1:** Specification of the proposed glucose biosensor.

No.	Parameter/Feature	Specification
1	Operating methodology	RF resonance frequency shifting
2	Applied material	PVA
3	Application	Glucose detection
4	Measurement range	10 µM to 1 M
5	Sensitivity	40.89 kHz/decade
6	Linearity	0.988
7	Response time	~3 s
8	Recovery time	~3 s
9	Repeatability	0.014%
10	Reproducibility	0.0135%

**Table 2:** Comparison of different glucose sensors.

No.	Operation Principle	Dynamic Range	Linearity	Response Time	Ref.
1	Resonance frequency shift	10 μM–1 M	0.988	~3 s	This work
2	Colorimetric	0.1–1.0 mM	-	-	[5]
3	Conductometric	0.5–12 mM	0.9602	Good	[6]
4	Fluorescence	0–500 μM	Not linear	-	[7]
5	Fiber grating	0–12 mM	-	20 min	[8]
6	Flexible RF sensor	0–80 mM	Good	-	[9]
7	Glucose-to-Resistor	0–12 mM	0.96	2 min	[10]
8	Hydrogel grating	0–50 mM	0.9724	-	[12]
9	Electrochemical	0.5–8 mM	-	5 s	[18]

## Data Availability

Data are contained within the article.
